# Self-Regulation and Executive Function Longitudinally Predict Advanced Learning in Preschool

**DOI:** 10.3389/fpsyg.2020.00049

**Published:** 2020-01-23

**Authors:** Steven James Howard, Elena Vasseleu

**Affiliations:** ^1^Early Start & School of Education, University of Wollongong, Wollongong, NSW, Australia; ^2^School of Psychology, University of Wollongong, Wollongong, NSW, Australia

**Keywords:** advanced learning, giftedness, executive function, self-regulation, school readiness

## Abstract

While the early years are often regarded as a critical period for establishing and supporting the developmental trajectories of delayed and typically developing children, they also represent a critical time for advanced learners. Yet to support advanced learners, a better understanding of sources and mechanisms of precocious early learning is needed. While there is ample research separately indicating importance of executive functions (EFs) and self-regulation for learning more broadly, it remains unclear whether, which, and to what extent EFs and/or self-regulation might account for the incidence of advanced learning in the prior-to-school years. The current study sought to investigate the EFs and self-regulation of 214 3- to 5-year old preschoolers, to better understand the profile of these abilities amongst advanced compared to non-advanced learners. Measures of self-regulation, EF and academic learning were taken at the start of the final pre-school year, and academic learning was assessed again at the end of the year. Results indicated that consistently advanced learning was predicted by socio-demographic factors (age, socioeconomic context), stronger cognitive development (combined EFs, cognitive aspects of self-regulation), yet lower behavioral self-regulation ratings. Results thus identify a profile of cognitive and behavioral characteristics of advanced early learners, which potentiates early identification and helps to clarify the nature and underpinnings of advanced early learning. It also raises questions about whether lower levels of behavioral self-regulation might constrain learning (e.g., difficulty remaining within the structures and sequences of the situation) or is a hallmark that is promotive of learning (e.g., convergent thinking, creativity).

## Introduction

It is widely acknowledged that the pre-school years are foundational for establishing and supporting the developmental trajectories of typically developing children, as well as children with developmental delay. It is also a critical time for advanced learners, for whom recognition and appropriate educational experiences can support their precocious development. Advanced early learners – similar to gifted children in the later school years – are often characterized by their strong and rapid knowledge acquisition (e.g., in language, reading, mathematics), good memory, keen interests, high attention to detail, deep levels of investigation and understanding, good problem solving, and strong self-motivation (Chamberlin et al., 2007; Cukierkorn et al., 2007). Yet the success of these learners is not assured, in the absence of appropriately supportive educational strategies. Where advanced learners are not recognized and supported, ineffectual or misdirected educational experiences place these children at risk of poorer educational, behavioral, social, and emotional outcomes (Gross, 1999; Hodge and Kemp, 2000; Pfeiffer and Stocking, 2000; Cukierkorn et al., 2007; Walsh et al., 2012). Conversely, where educational strategies are tailored to the learner’s advanced abilities (e.g., acceleration of advanced learners into kindergarten to accommodate their intellectual needs), their outcomes are similar to or better than their older classmates (e.g., yielding improvements in school adjustment, enthusiasm for learning, self-efficacy; Daurio, 1979; Robinson and Weimer, 1991; Walsh et al., 2012).

While there are some effective early intervention approaches for advanced early learners (Henderson and Ebner, 1997; Walsh et al., 2012), there is need for a better understanding of the sources and mechanisms of advanced early learning. This would aid the identification of viable targets for intervention (e.g., teaching content-specific learning such as early numeracy, and/or supporting the content-free underpinnings of learning) and promote consistency in intervention outcomes. Previous studies of these mechanisms often point to cognitive control processes that facilitate effective and efficient learning. Specifically, evidence of higher levels of executive functions (EFs) amongst advanced learners (Johnson et al., 2003; Bracken and Brown, 2006; Arffa, 2007), as well as the established role of EFs in learning more generally (Bull et al., 2008; Clark et al., 2010; Fuhs et al., 2014), suggest EFs as a possible mechanism for these rapid rates of knowledge acquisition. In this context, better EFs permit: concurrent activation and processing of greater quantities and complexities of information in mind (working memory); resistance to impulses, distractions, and irrelevant information that could detract from learning (inhibition); and the ability to flexibly apply and shift attention with changing demands of the situation and intended learning outcomes (shifting).

It is unclear whether this advantage might stem from superior EF *per se* (a higher capacity in one or more EFs), or from more-effective EF-mobilization strategies (e.g., information processing strategies, problem solving strategies; Ball et al., 1994; Johnson et al., 2003). While effective EF-mobilizing strategies often accompany high EF capacities (Roebers and Feurer, 2016), this is not necessarily so. For instance, previous research suggests cognitively gifted students may be characterized by an advantage in their endogenous EF capacity and/or experiential developmental factors (e.g., learned executive know-how, such as strategies that reduce the executive demands of a task; Johnson et al., 2003; Howard et al., 2013). Accordingly, gifted students’ advanced performance may be facilitated, at least in some cases, by more effective learned strategies for deploying EF resources rather than more-rapid development of their endogenous capacities.

There is also some evidence that advanced early learners may have higher levels of self-regulation (Calero et al., 2007), further supporting the possibility that advanced early learning may be better characterized by acquired and more-malleable cognitive control strategies (in contrast to EF capacities, which have proven resistant to broadly transferrable improvements; Diamond and Ling, 2016). To explain the relationship between EF and self-regulation, Hofmann et al. (2012) position EFs as the capacity component of self-regulation, which are dynamically and contextually integrated with goal-setting, motivation, and problem-solving to achieve successful self-regulation. According to this framework, successful learning (as in the case of advanced learners) would be the product of: pursuing a learning objective (goal setting); persisting with this until its conclusion (motivation), even if this becomes difficult (problem solving), as well as the ability to direct sufficient cognitive resources toward learning (capacity, or EFs). In this model, EFs are a necessary but not sufficient condition for successful self-regulation.

However, findings concerning the self-regulation of advanced early learners are mixed, with some findings of increased behavioral problems amongst gifted students. For instance, in one study, similar behavioral profiles were found for gifted children and children diagnosed with ADHD (e.g., similarly high rates of oppositional and hyperactive behaviors; Alloway and Elsworth, 2012). Indeed, there is considerable overlap in the behaviors associated with ADHD and giftedness (Webb and Latimer, 1993). Reasons for this apparent disconnect between advanced cognition and problem behaviors are unclear, with suggestions ranging from: over-excitability being interpreted as hyperactivity; disruptive behaviors that arise from boredom, due to a lack of cognitive challenge (Alloway and Elsworth, 2012); or comparatively lower levels of impulse control amongst gifted students (Johnson et al., 2003).

Understanding whether, which and to what extent EFs and/or self-regulation can account for the incidence of advanced learning in the prior-to-school years is complicated by the fact that EF and self-regulation have tended to be studied in isolation of each other, with little integration in the context of advanced learning. It is also unclear whether advanced learners’ profile of performance in these areas should be expected to be uniformly high, or rather might reveal a more nuanced profile of developmental strengths and opportunities. The current study thus sought to investigate preschoolers’ EFs and self-regulation together, to better understand the concurrence and profile of these abilities amongst advanced early learners compared to non-advanced early learners. In line with preliminary evidence that advanced early learning might be better characterized by more effective strategies for mobilizing cognitive resources toward learning, it was expected that measures indexing complex integration and application of EFs (e.g., a multi-faceted EF task, cognitive self-regulation index), rather than isolated EFs, would provide comparatively better prediction of advanced early learning when concurrently modeled. Further, in line with findings of more prevalent behavior problems amongst young gifted children (although see Richards et al., 2003 for contradictory findings), it was also expected that advanced early learners would be comparatively lower in behavioral self-regulation. It was anticipated that findings would further clarify the mechanics and mechanisms of advanced learning in the early years, and thereby suggest viable targets and approaches to appropriately support young advanced learners.

## Materials and Methods

### Participants

All children attending one of the 25 participating pre-school centers in metropolitan and regional areas of Australia, identified by their parents as likely attending school the following year, were invited to participate in this study. Centers were selected to be broadly representative of population proportions in terms of their geography (84% metropolitan), socio-economic decile for their catchment area (*M* = 5.91, *SD* = 2.24, range = 1–10), and statutory quality assessment rating (i.e., 44% Exceeding, 48% Meeting, 4% Working Toward, 4% unrated against the National Quality Standard).

Parental consent to participate was provided for 217 3-5-year old children, all of whom were identified as likely to be attending school in the subsequent year. While in the Australian context it is most common for children to commence school at age 5, this does not preclude children from commencing younger, as reflected in the smaller number of 3-year-olds (*n* = 29, with only two younger than 3.5 years) in the sample who were identified as starting school the next year. At baseline, the mean age of the sample was 4.43 years (*SD* = 0.38, range = 3.20–5.24), with a relative balance of boys and girls (46.5% girls). Children who identified as of Aboriginal or Torres Strait Islander descent comprised 5.2% of the sample, which is in line with population estimates for this age group (Australian Institute of Health and Welfare [AIHW], 2012). Family income was diverse: 10.6% of families qualified for full childcare benefit subsidies (low income); 65.4% of families qualified for some childcare benefit (low-middle to middle-high income); and 24.0% of families did not qualify for any childcare benefit subsidy (high income). Maternal education levels were also diverse: 9.1% did not complete high school; 8.0% completed only high school; 29.9% had completed a diploma, trade, or certificate; 34.8% completed a tertiary degree; and 18.2% a post-graduate qualification. All children spoke English as their first language. This study was approved by the University of Wollongong’s Human Research Ethics Committee, and participants were those whose parents provided informed written consent and themselves provided verbal assent to participate.

### Measures

#### Academic Learning

The academic knowledge of participating children was assessed using the *Bracken School Readiness Assessment (BSRA*, 3rd edition; Bracken, 2007). BSRA is a standardized assessment of areas deemed important for school readiness. It includes subscales of colors (10 items), letters (15 items), numbers/counting (18 items), sizes/comparisons (22 items), and shapes (20 items). For each domain, the assessment continues until completion or three consecutive incorrect responses. BSRA has been shown to be predictive of kindergarten teacher ratings of children’s school readiness and academic results (Bracken, 2007; Panter and Bracken, 2009). Children’s rate of academic learning was examined using multiple BSRA indices, namely: children’s raw scores, to evaluate change with age; standard scores, to evaluate change in relative age-adjusted terms; and, finally, established performance thresholds to classify learners as “delayed to average” or “advanced to very advanced”. Validity of these classifications is shown through their prediction of clinical diagnoses (e.g., language delay or disorder) and later outcomes (Bracken, 2007).

#### Executive Functions

Individual EFs were indexed by measures of working memory, inhibition, and cognitive flexibility selected from the iPad-based *Early Years Toolbox (EYT*; Howard and Melhuish, 2017). Specifically, working memory was indexed by the *Mr. Ant* task, which asks children to remember the spatial locations of “stickers” placed on a cartoon ant, and identify these locations after a brief retention interval. Test trials increase in complexity as the task progresses (progressing from one to eight stickers), with three trials at each level, until the earlier of completion or failure on three trials at the same level of difficulty. Working memory was indexed by a point score that estimates working memory capacity, following protocols of Howard and Melhuish (2017). Inhibition was assessed by the *go/no-go* task, which requires participants to respond to “go” trials (“catch fish”) and withhold responding on the “no-go” trials (“avoid sharks”). The majority of stimuli are “go” trials (80% fish), thereby generating a pre-potent tendency to respond that children must inhibit on “no-go” trials (20% sharks). After instruction and practice, 75 test stimuli were presented across three 1-min blocks (separated by a short break and reiteration of instructions). Each trial involved presentation of an animated stimulus (i.e., fish or shark) for 1500 ms, each separated by a 1000 ms inter-stimulus interval. In line with protocols of Howard and Melhuish (2017), inhibition was indexed by an impulse control score, which is the product of proportional “go” (to account for the strength of the pre-potent response generated) and “no-go” accuracy (to index a participant’s ability to overcome this pre-potent response). Finally, cognitive flexibility was assessed by the *Card Sort* task, which asks children to sort cards (i.e., red rabbits, blue boats) first by one sorting dimension (e.g., color), then switch to the other sorting dimension. The task begins with a demonstration and two practice trials, after which children begin sorting by one dimension for six trials. In the subsequent post-switch phase, children are asked to switch to the other sorting dimension. For all test items, each trial begins by reiterating the relevant sorting rule and then presenting a stimulus for sorting. If the participant correctly sorts at least five of the six pre- and post-switch stimuli, they then proceed to a border phase of the task. In this phase, children are required to sort by color if the card has a black border or sort by shape if the card has no black border. Cognitive flexibility was indexed by the number of correct sorts after the pre-switch phase (Howard and Melhuish, 2017). Each of these tasks has shown good convergent validity with other task-based measures of EF (*r*s ranging from 0.40 to 0.46) and reliability with children of this age (Howard and Melhuish, 2017).

A measure that requires complex combination of EFs was also administered. *Head-Toes-Knees-Shoulders (HTKS)* asks children to remember a correspondence between body parts (e.g., head and knees), and then perform the opposite action to what was indicated (e.g., touch their knees when the facilitator says “touch your head”). In doing so it requires children to hold a correspondence in mind (working memory), inhibit the impulse to do as directed (inhibition), and flexibly switch between correspondences across task levels (cognitive flexibility). The task consists of six practice and 10 test trials at each of three levels of difficulty: (1) correspondence between head and toes; (2) correspondence between knees-shoulders and head-toes; and then (3) flexibly switching between the correspondences of head-knees and shoulders-toes. The task continues until completion or failing to achieve at least four points within a level (such that two points are awarded for a correct response and one point for a self-corrected correct response). HTKS has been shown to have good convergent validity with other task- and adult-report measures of self-regulation, predictive validity of academic learning, and psychometric reliability (e.g., α ranging from 0.92 to 0.94; McClelland et al., 2014). Fieldworkers completed the online training module prior to in-field data collection to ensure accuracy of scoring and inter-rater reliability. Performance was indexed by the sum of points awarded across all practice and test trials.

#### Self-Regulation

*Preschool Situational Self-Regulation Toolkit (PRSIST) Assessment* (Howard et al., 2019) is an observational measure of early self-regulation that engages children in self-regulatory activities, and rates the child’s behavior in each activity in relation to key aspects of cognitive and behavioral self-regulation. The first PRSIST Assessment activity is a group memory card game. In this activity children, in a group of four, take turns trying to find a matching pair of cards (e.g., 8 pairs for 4-year-olds, 14 pairs for 5-year-olds), which takes around 10 min to complete. The second activity is an individual curiosity boxes activity, in which children are presented with a series of three boxes of increasing size and they are asked to guess their contents. The sequence of guessing occurs as follows: first, guess based only on the size of the box (no touching); second, guess after gently lifting the box to feel its weight (no shaking); third, guess after shaking the box (no opening); and lastly, guess after closing your eyes and feeling the object inside (no peeking). This takes around 5 min to complete. Rather that considering the number of pairs found or correct guesses, however, performance is rated by a trained observer in terms of each child’s self-regulatory behaviors. Specifically, each child’s self-regulation is rated at the end of each activity, with items rated along a 7-point Likert scale representing a judgment of the frequency and/or degree of behaviors relating to cognitive self-regulation (e.g., Did the child sustain attention, and resist distraction, during the instructions and activity?) and behavioral self-regulation (e.g., Did the child control their behaviors and stay within the rules of the activity?). This yielded two sets of self-regulation ratings per child – one per activity – which were averaged for the two activities before aggregating into cognitive (six items) and behavioral self-regulation indices (three items). To ensure inter-rater reliability, each of the four fieldworkers completed the online training module^1^. This was followed by: five joint observations alongside a member of the research team prior to in-field data collection; and inter-rater reliability checks, in which all raters achieved a minimum correlation between ratings greater than *r* = 0.70, a mean difference in ratings less than 0.75 points and at least 80% of item ratings within 1 point. This measure has shown good construct validity, reliability (α ranging from 0.86 to 0.95), and concurrent validity with task-based self-regulation (*r*s ranging from 0.50 to 0.63) and school readiness measures (*r*s between 0.66 and 0.75) (Howard et al., 2019).

#### Demographics

##### Demographic covariates

Parents reported on demographic information used as covariates for analyses. These were: child’s age (the date of assessment minus date of birth); child’s sex (1 = male, 2 = female); the Australian Bureau of Statistics [ABS] (2012) Socio-Economic Indexes for Areas (SEIFA), which is a postcode-level index of socioeconomic decile created by the ABS by combining census data on factors such as education, household income, and unemployment. This area-level index was used over the family income variable given its increased sensitivity (reported in deciles) over the three wide income bands utilized to capture eligibility for childcare benefit.

### Procedure

All tasks were administered to children in a quiet area of their pre-school center in five sessions across the same day, to maximize children’s attention and minimize fatigue. Measures were administered in the same order to all children, as follows: (1) BSRA; (2) PRSIST curiosity boxes and HTKS; (3) Mr Ant and Go/No-Go; (4) PRSIST memory; and (5) Card Sort. Each session took 10–20 min to complete, and were done near the start of children’s final pre-school year (March–April 2018). PRSIST raters were not involved in BSRA administration and were blind to EF and BSRA scores at the time of rating. BSRA was again conducted near the end of the year (October–November 2018), also in a quiet area of the child’s pre-school center.

### Analytic Approach

Multinomial logistic regression was used to examine the associations of children’s start-of-year cognitive and demographic data with end-of-year learner classifications. To do this, all participants were categorized using BSRA standard (age-adjusted) scores and classifications as: (1) “not advanced,” on the basis of being at or below age expectations at both time points; (2) “no longer advanced,” on the basis of children’s scores being “advanced” or “very advanced” at the start of year, but at or below age expectations at the end of the year; (3) “newly advanced,” on the basis of being at or below age expectations at the start of the year and advanced at the end of the year; or (4) “consistently advanced”, on the basis of showing advanced performance at both time points. The referent group for all multinomial regression analyses was the “not advanced” group, to investigate characteristics that differentiated advanced learners from those consistently at or below age expectations.

## Results

### Initial Data Exploration

Initial data exploration indicated that one child (0.5%) did not complete the Card Sort task due to early departure on the day of assessment and 27 parents (12.4%) declined to provide their postcode for purposes of SES estimation. In these cases, the modal SEIFA decile for the preschool catchment area was used (which, in the majority of cases, corresponded to the sole SEIFA decile for children attending that service). This resulted in loss of only one data point. Subsequent data exploration indicated that the assumptions for multinomial logistic regression were met for the analytic sample. Specifically, in a linear regression predicting BSRA standard scores, despite a strong correlation between PRSIST subscales (and modest associations for other predictors; Table 1), all potential predictors showed VIFs well below 10 (range = 1.04–2.61), thereby justifying their concurrent inclusion in multinomial logistic regression analysis.

**TABLE 1:** Correlations amongst continuous predictors and Bracken School Readiness Assessment (BSRA) standard score.

		**1**	**2**	**3**	**4**	**5**	**6**	**7**	**8**	**9**
1	Age (T1)	−	−0.20*	–0.05	0.29*	0.33*	0.30*	0.29*	0.15*	0.28*
2	BSRA		−	0.17*	0.35*	0.26*	0.10	0.25*	0.19*	0.33*
3	SEIFA			−	0.01	0.22	–0.13	0.02	–0.06	–0.06
4	HTKS				−	0.39*	0.35*	0.36*	0.34*	0.38*
5	PRSIST_C					−	0.75*	0.48*	0.34*	0.39*
6	PRSIST_B						−	0.43*	0.36*	0.24*
7	Mr Ant							−	0.30*	0.37*
8	Go/No-Go								−	0.19*
9	Card Sort									−

Age at baseline is analyzed here, given this is the age at which these assessments were taken. BSRA represents standard (age-adjusted) scores used for subsequent classification and analyses. *p < 0.05.

### Prevalence of Learner Groups

As expected, prevalence rates for each learning group were consistent with theoretical estimates (i.e., 10–15%) of the prevalence of advanced/gifted learners (Gagne, 2003). That is, at baseline there were 35 (16.1%) children identified as advanced or very advanced in academic knowledge by their BSRA scores. At end-of-year follow-up, there were 28 children (12.9%) whose performance identified them as advanced or very advanced. Yet children did not always remain in their initial category: 172 children (79.3%) were not advanced at either time point; 17 children (7.8%) were advanced at baseline, but no longer at follow-up; 10 children (4.6%) were not advanced at baseline, but were at follow-up; and 18 children (8.3%) were consistently advanced at both time points. An evaluation of raw BSRA scores suggested that this reflected a difference in the *rates* of knowledge acquisition, as raw scores improved or remained stable across all four groups (see Table 2). As such, this pattern demonstrated differing trajectories of learning amongst the sample; while some children started and remained high (or average-to-low) in academic knowledge, other students showed a slower rate of learning (i.e., started high, but a slow-to-nil rate of change over the year meant they were no longer advanced for their age by year end) or a faster rate of learning (i.e., started average-to-low but showed a rapid rate of knowledge acquisition that led to them being advanced for their age by end of year). While the “not advanced” group included a small number of children who were very delayed (*n* = 5) or delayed (*n* = 29) at baseline, a number of these children (*n* = 16) improved to average by the end of the year. As such, to best capture the full range of school readiness in pre-school settings, these children were retained for analyses. Patterns of results did not differ with their exclusion.

**TABLE 2:** BSRA raw and standard scores by learner group.

Learner group	T1 Raw score M (SD)	T2 Raw score M (SD)	T1 Std. score M (SD)	T2 Std. score M (SD)
Not advanced	43.79 (13.95)	55.19 (13.55)	94.47 (11.30)	95.17 (11.42)
No longer	66.35 (6.41)	67.94 (6.36)	117.35 (2.45)	107.69 (4.30)
Newly	52.20 (14.76)	72.70 (5.91)	104.30 (7.23)	117.80 (2.94)
Consistently	67.06 (6.98)	75.39 (4.38)	121.56 (5.26)	122.83 (5.32)

*Not advanced is the reference category, referring to the majority of children who were at or below age expectations on BSRA at both time points. No longer refers to children who were advanced for their age on BSRA at baseline, but not at follow-up. Newly refers to children who were not advanced for their age at baseline, but were at follow-up. Consistently refers to children who were advanced on BSRA for their age at both baseline and follow-up. T1, baseline (start of year). T2, follow-up (end of year). Std. Score, Bracken School Readiness Assessment’s age-adjusted school readiness score.*

### Predictors of Early Advanced Learning

The presence of these differing learning trajectories justified subsequent analyses, which examined self-regulatory, EF, and demographic predictors of each advanced learning group, relative to children who were not advanced at either time point. Descriptive statistics of these predictors are provided in Table 3. Results of the multinomial logistic regression (see Table 4) indicated that few variables significantly predicted differences for the “no longer advanced” or “newly advanced” group compared to the “not advanced” group. Children within the “no longer advanced” group were more likely to be male than female compared to the not advanced group (*RRR* = 0.23, 95% CI = 0.07 to 0.80, *p* = 0.021). Children in the newly advanced group were more likely to reside in higher-SES areas than the not advanced group (*RRR* = 1.60, 95% CI = 1.11 to 2.32, *p* = 0.013). No other predictors achieved significance, although this should be considered in relation to the relatively small cell sizes for these groups (i.e., 17 and 10 children).

**TABLE 3:** Descriptive statistics for baseline predictors by learner group.

	Not advanced M (SD)	No longer M (SD)	Newly M (SD)	Consistently M (SD)
**Demographics**				
Age (Time 2)	5.01 (0.37)	5.01 (0.38)	4.92 (0.44)	4.77 (0.44)
SEIFA decile	5.80 (2.18)	6.47 (1.84)	7.50 (2.01)	7.22 (1.80)
**Executive function**				
HTKS	20.69 (22.28)	31.06 (30.42)	32.80 (31.88)	32.72 (26.37)
Mr Ant (WM)	1.49 (0.94)	1.86 (0.79)	1.67 (1.28)	1.70 (0.94)
Go/No-Go (Inh)	0.56 (0.19)	0.66 (0.21)	0.63 (0.19)	0.58 (0.12)
Card Sort (CF)	4.34 (4.08)	6.53 (4.42)	4.40 (4.22)	6.22 (3.67)
**Self-Regulation**				
PRSIST (CSR)	3.21 (1.21)	3.64 (1.33)	3.33 (1.52)	3.47 (0.97)
PRSIST (BSR)	4.33 (1.21)	4.49 (1.00)	4.15 (1.54)	3.89 (1.17)

*Not advanced is the reference category, referring to the majority of children who were at or below age expectations on BSRA at both time points. No Longer refers to children who were advanced for their age on BSRA at baseline, but not at follow-up. Newly refers to children who were not advanced for their age at baseline, but were at follow-up. Consistently refers to children who were advanced on BSRA for their age at both baseline and follow-up. SEIFA, Australian Bureau of Statistics’ area-level Socioeconomic Indices for Areas, derived from postcode-level socioeconomic census data. HTKS, head-toes-knees-shoulders task; WM, working memory; Inh, inhibition; CF, cognitive flexibility; PRSIST, preschool situational self-regulation toolkit assessment; CSR, cognitive self-regulation; BSR, behavioral self-regulation.*

**TABLE 4:** Association of predictors with children’s learner group membership.

	No longer vs. Not advanced			Newly vs. Not advanced			Consistently vs. Not advanced		
	Exp(B)	95% CI	*p*	Exp(B)	95% CI	*p*	Exp(B)	95% CI	*p*
Age (Time 2)	0.19	0.03–1.15	0.071	0.16	0.02–1.41	0.099	**0.02**	0.00–0.17	<0.001
Sex	**0.23**	0.07–0.80	0.021	0.32	0.07–1.46	0.141	0.56	0.17–1.86	0.340
SEIFA	1.27	0.97–1.67	0.084	**1.60**	1.11–2.32	0.013	**1.53**	1.13–2.08	0.006
HTKS	1.01	0.99–1.03	0.379	1.03	0.99–1.06	0.104	**1.03**	1.00–1.06	0.035
Mr Ant	1.01	0.49–2.09	0.981	0.95	0.37–2.45	0.916	1.39	0.61–3.20	0.436
Go/No-Go	16.47	0.57–478.35	0.103	6.40	0.09–436.14	0.389	1.24	0.03–47.66	0.910
Card Sort	1.15	0.99–1.34	0.068	1.00	0.82–1.21	0.999	1.15	0.97–1.36	0.113
PRSIST (CSR)	1.45	0.71–2.97	0.309	1.73	0.65–4.64	0.273	**3.62**	1.49–8.80	0.005
PRSIST (BSR)	0.79	0.38–1.64	0.522	0.58	0.22–1.55	0.280	**0.28**	0.12–0.66	0.004

*Exp(B) indicates the relative risk ratio, which can be broadly interpreted as the proportional increase in relative risk/chance of the outcome (being in a given advanced learner group) with a one-unit change in the predictor variable. Significant relative risk ratios are identified in bold. Not advanced is the reference category, referring to the majority of children who were at or below age expectations on BSRA at both time points. No Longer refers to children who were advanced for their age on BSRA at baseline, but not at follow-up. Newly refers to children who were not advanced for their age at baseline, but were at follow-up. Consistently refers to children who were advanced on BSRA for their age at both baseline and follow-up. SEIFA, Australian Bureau of Statistics’ area-level Socioeconomic Indices for Areas, derived from postcode-level socioeconomic census data. HTKS, head-toes-knees-shoulders task; PRSIST, preschool situational self-regulation toolkit assessment; CSR, cognitive self-regulation; BSR, behavioral self-regulation.*

In contrast, a broad range of factors significantly differentiated the consistently advanced learner group from the not advanced group (Table 4). Specifically, the consistently advanced group was characterized as: having higher scores on HTKS (*RRR* = 1.03, 95% CI = 1.00 to 1.06, *p* = 0.035), but not on individual EF tasks (working memory: *p* = 0.436; inhibition: *p* = 0.910; cognitive flexibility: *p* = 0.113); cognitive self-regulation (*RRR* = 3.62, 95% CI = 1.49 to 8.80, *p* = 0.005) and behavioral self-regulation (*RRR* = 0.28, 95% CI = 0.12 to 0.66, *p* = 0.004); being younger (*RRR* = 0.02, 95% CI = 0.00 to 0.17, *p* < 0.001); and living in higher-SES areas (*RRR* = 1.53, 95% CI = 1.13 to 2.08, *p* = 0.006). Child sex was not a significant predictor of being in the consistently advanced group compared to the not advanced group (*p* = 0.340). Given that all variables were included in the regression simultaneously, this indicates unique and independent prediction of each of these factors even after controlling for all other included variables. As a final step, analyses were replicated for all children who were advanced at follow-up (i.e., consistently and newly advanced children) referenced to the not advanced group. The pattern of results was maintained, such that significant predictors of being in the advanced group were: age, *RRR* = 0.05, 95% CI = 0.01 to 0.24, *p* < 0.001; SEIFA, *RRR* = 1.55, 95% CI = 1.21 to 1.99, *p* = 0.001; HTKS, *RRR* = 1.03, 95% CI = 1.01 to 1.05, *p* = 0.013; cognitive self-regulation, *RRR* = 2.70, 95% CI = 1.35 to 5.42, *p* = 0.005; and behavioral self-regulation, *RRR* = 0.38, 95% CI = 0.19 to 0.76, *p* = 0.006.

## Discussion

The current study sought to investigate the cognitive and behavioral profile of advanced academic learners in the pre-school years. Results from the longitudinal analysis of start- and end-of-year data identified that advanced learning was predicted by socio-demographic factors (i.e., age, socioeconomic context), cognitive factors (i.e., combined EFs, cognitive aspects of self-regulation) and behavioral factors (i.e., behavioral facets of self-regulation). While for most predictors advanced learners showed an advantage in these abilities, they also showed significantly lower levels of behavioral aspects of self-regulation. These results identify a profile of cognitive and behavioral characteristics of early advanced learners, which potentiates early identification and helps to clarify the nature and possible underpinnings of early advanced learning.

The socio-demographic factors that were associated with end-of-year advanced learning were the child’s age and socioeconomic context. Age was negatively associated with advanced learning, such that younger children were significantly more likely to have advanced academic knowledge (of letters, colors, shapes, numbers, sizes). While this may seem counterintuitive, this finding must be considered in the context of the age-relative nature of this classification and narrow age range of the current sample. That is, school readiness standard scores identify children’s academic learning progress relative to their age, such that a young child who has an identical score to an older child will be characterized as comparatively more advanced in their academic knowledge, for their age. Further, all children in the current sample were identified by their parents as likely to be attending school the following year. Younger children are more likely to be accelerated into school if they are advanced in their learning relative to age peers, whereas comparatively older children with lower levels of readiness for transition are more likely to be kept in preschool for another year. This was in line with the current result, wherein younger children in their final pre-school year were more advanced in their learning than older children after age standardization of BSRA scores.

That a child’s socioeconomic context also predicted advanced levels of learning was in line with previous findings of a socio-economic gradient for academic achievement (Considine and Zappala, 2002). Specifically, Considine and Zappala (2002) found that school performance was predicted by a range of social factors (e.g., unexplained school absences, child gender) and economic factors (e.g., parental education, housing). Indeed, a meta-analysis of more than 100,000 students indicated a moderate to strong SES effect on academic achievement (Sirin, 2005). It is thus unsurprising that age and SES were strong predictors of whether or not a child was an advanced learner in the current study, over and above the variability accounted for by cognitive and behavioral indices. The lack of national curriculum in Australia, which is instead governed by an *Early Years Learning Framework*, suggests this is not merely a proxy for high-quality preschool provision or curricula. Indeed, there was little clustering of advanced learners within centers. Among the 25 participating centers, one center had four persistently advanced learners (of nine participating children at that center), one center had three advanced learners (out of nine children), two centers had two advanced learners (out of 15 children), and seven centers had one advanced learner (out of 60 children).

There is also ample research suggesting an advantage in EFs – and especially working memory – amongst gifted learners (Johnson et al., 2003; Arffa, 2007; Howard et al., 2013). A study by Visu-Petra et al. (2011) highlighted the role of EFs across the spectrum of learners, such that individual differences in EF accounted for 50% of the variability in students’ academic performance (see also van den Bos et al., 2013; Shaul and Schwartz, 2014). However, the current results suggest that it may not be individual and isolated EF capacities *per se*, but rather their effective combination, mobilization and application in real-world contexts (e.g., paying and sustaining attention during learning experiences, cognitive engagement in learning tasks, ability to be self-directed) that are better predictors of advanced learning. While there is general consensus that EFs are involved in cognitive and behavioral self-regulation – such as using working memory resources to maintain goals in mind, inhibiting distractions, and flexibly deploying attention in service of one’s goals (as was the case for the HTKS task) – the two are not synonymous. In an educational context, for instance, failure to acquire new learning can result from never deciding to invest energy toward learning (goal setting), giving up early (motivation), having insufficient strategies to overcome barriers to learning (problem-solving strategies), or insufficient cognitive resources to concentrate on and work with targets of learning (capacity). Only the latter pertains to EFs, although self-regulatory failure can arise from a failure in any of these aspects. This does not contradict or diminish the role of EFs in learning, or as a characteristic advantage amongst advanced learners. Rather, it suggests that the EF advantage of advanced learners may more accurately be characterized as more effective combination, application, and integration of EFs within complex cognitive undertakings, in the presence of sufficient (high) EF capacity for the task at hand.

While the cognitive dimension of self-regulation was higher amongst advanced learners in the current study, these children were also characterized by lower levels of behavioral self-regulation. However, this finding should be interpreted in light of the dimensions of behavioral self-regulation that were assessed, namely: controlling behaviors to remain within the rules and requirements of the activity; remaining seated, and not overly fidgeting; and following social expectations of the activity (e.g., taking turns, not talking over others, acknowledging others’ successes). There are multiple plausible explanations for this discrepant profile of abilities. For instance, for advanced learners with high cognitive self-regulation, requirements such as waiting for others who may require additional time (or, rather, may be more impulsive and may be rushing the advanced learner while they consider their options) may be especially difficult. Lower levels of behavioral self-regulation thus may be a consequence of advanced learning, as noted in prior studies that indicate slow pace and repetition as sources of boredom, frustration and underachievement for gifted children (Baker, 1996; Gallagher et al., 1997). It may additionally be that behaviors associated with high achievement are misconstrued as behavioral dysregulation, such as in cases where “off-topic” questions from gifted students are dismissed by educators (Vialle and Rogers, 2009), even though the question is perfectly on topic but takes a creative interpretation or is a number of steps ahead (as illustrated by the following question from a preschooler: “If a dog had six legs, would it run faster?”; Vialle and Rogers, 2009, p. 33). As the current study is not able to determine between these options, this remains a worthwhile area for further investigation.

While the robustness of the current results is supported by the relatively large and diverse sample, longitudinal data, and multiple measures, there are nevertheless limitations that qualify these findings. For instance, low cell sizes (e.g., for no longer advanced learners) precluded a comprehensive evaluation of the characteristics associated with changes in learning trajectory. Further investigations in this area could identify targets for intervention or prevention to ensure all students are achieving to their potential. At present, the reasons for these changes in group membership are unclear (e.g., regression to the mean, environmental precursors; Gross, 1999). Further, the current analysis presumes four categories of learner, but more are plausible (e.g., very delayed, delayed, average, advanced, very advanced). Larger and more longitudinal data sets would be required to investigate the merits of these classifications, and how characteristics might change across them. Lastly, given the focus of the current study was on integrating EF and self-regulation data, a non-exhaustive range of additional factors were considered. There are other plausible environmental factors (e.g., child’s attendance at high-quality preschool, home learning environment) that could be expected to exert similar or greater influence.

Notwithstanding these limitations, the current study suggests an interesting profile for consistently advanced early learners, in terms of cognitive strengths (i.e., coordination and application of EFs to complex cognitive tasks) and aspects of behavioral regulation that were seemingly not as strong. This is in contrast to previous findings that imply that EF capacities appear greater amongst advanced learners–capacities that are notoriously difficult to shift in a way that achieves flow-on benefits to real-world outcomes. Instead, the current results suggest that it may instead (or in addition) be that more malleable aspects of performance are contributing to incidence of advanced learning, suggesting the possibility that these strategies might be fostered for the benefit of more learners. In demonstrating a non-uniform profile of development for advanced learners, these results also raise questions that warrant further study. For instance, while it is clear that advanced learners in the current sample were lower in behavioral aspects of self-regulation, it remains unclear whether this was a factor constraining their learning (e.g., difficulty remaining within the structures and sequences of the situation) or rather a hallmark of their manner of engagement in/with learning (e.g., convergent thinking, creativity). The current study thus represents a clarification and stimulus for further research into the nature of early learning and characteristics of highly effective early learners.

## Data Availability Statement

The dataset for this article is not publicly available because ethics approval was not sought or granted for such use. Requests to access the dataset should be directed to SH at stevenh@uow.edu.au.

## Ethics Statement

This study was approved by the University of Wollongong’s Human Research Ethics Committee, and participants were those who provided written parental consent and themselves provided verbal assent to participate.

## Author Contributions

SH conceptualized the study, secured funding for the study, oversaw data collection, analyzed the data, and led writing of the manuscript. EV aided in conceptualizing the study, managed the data collection and entry, and contributed to drafting of the manuscript.

## Conflict of Interest

The authors declare that the research was conducted in the absence of any commercial or financial relationships that could be construed as a potential conflict of interest.

## Footnotes

1 http://www.eytoolbox.com.au
